# Neuroticism, extraversion, and emotion sensitivity in a nonclinical young adult sample

**DOI:** 10.1371/journal.pone.0349027

**Published:** 2026-06-03

**Authors:** Lauren A. Rutter, Prabhvir Lakhan, Laura Germine

**Affiliations:** 1 Department of Psychological and Brain Sciences, Indiana University, Bloomington, Indiana, United States of America; 2 Indiana University School of Medicine, Bloomington, Indiana, United States of America; 3 Department of Neurology, Albert Einstein College of Medicine, Bronx, New York, United States of America; University of Reading - Whiteknights Campus: University of Reading, UNITED KINGDOM OF GREAT BRITAIN AND NORTHERN IRELAND

## Abstract

**Introduction:**

Accurate recognition of others’ emotional facial expressions, referred to as emotion sensitivity (ES), is a central component of nonverbal communication. Previous research suggests that personality traits such as neuroticism and extraversion may shape nonverbal emotion decoding but findings have been mixed. The present study examined whether these traits predict performance on the Belmont Emotion Sensitivity Test (BEST), a task designed to measure subtle discrimination of anger, fear, and happiness while minimizing response bias.

**Method:**

A sample of 289 college students completed self-report measures of neuroticism and extraversion, as well as the Belmont Emotion Sensitivity Test (BEST), which assesses the ability to discriminate subtle differences in facial expressions of fear, anger, and happiness. Depression, anxiety, and stress levels were also measured.

**Results:**

Female participants reported higher levels of neuroticism (*t* = 4.39, *p* < .001; *d* = .59) and extraversion (*t* = 2.33, *p* = .02; *d* = .33) *t*han male participants. Neuroticism severity was positively correlated with depression, anxiety, and stress. Extraversion was negatively correlated with depression, anxiety, and stress (range of *r*s = .55−.68, *p* < .001). Multiple regressions showed that neither neuroticism nor extraversion significantly predicted ES scores for any emotion category.

**Discussion:**

Although women reported higher levels of neuroticism and extraversion, these traits did not predict performance on a validated emotion sensitivity task. These findings suggest that, in this nonclinical young adult sample, neuroticism and extraversion were not robust predictors of performance on this measure of emotion sensitivity. Implications are discussed for theories linking personality, psychopathology, and nonverbal emotion perception.

## Introduction

The ability to decode others’ emotional facial expressions is a central component of communication. Individuals with emotional disorders have repeatedly demonstrated difficulties in perception of others’ emotions through facial expressions [1] and show other affective and cognitive biases [2,3]. Emotion perception is a multifaceted process involving appraisal and identification of emotions, production of an affective state in response, and regulating that affective state and emotional behavior (see [4] for a critical review). Here, we focus on one facet of emotion perception known as facial emotion sensitivity (ES), or, the ability to distinguish intensities of emotional facial expressions. We define ES as the ability to discriminate differences in emotional intensity, which differs from emotion recognition, a skill that involves identifying or labeling facial expressions. ES is a nonverbal decoding skill critical to social interaction. It has been linked to better quality of life and interpersonal functioning [5,6].

While some of the existing literature has produced mixed and contradictory results regarding facial emotion recognition abilities [7,8], in general, adults with anxiety disorders have shown moderate impairment in facial emotion recognition [9], while adults with depressive disorders have demonstrated difficulty in detecting happiness [10], with significant differences between genders (females show enhanced facial emotion recognition abilities relative to males [11–14]. In a sample of nearly 10,000 individuals aged 10–85, prior work demonstrated that ES abilities change across the lifespan based on emotion category (fear, happiness, anger) [15]. Prior work has shown that ES abilities are linked to symptom severity across mood and anxiety disorders. In anxiety disorders such as generalized anxiety disorder (GAD), more severe GAD scores are associated with poorer ES performance for all emotion categories (happiness, anger, fear) [16]. Prior work has also shown that depression severity is associated with lower ES performance across emotion categories [17]. Additionally, findings have demonstrated that posttraumatic stress symptom severity is associated with impaired ES performance, particularly for fear [18]. While these findings highlight the importance of ES in mental health, less is known about whether variations in personality traits predict similar differences in nonverbal decoding in healthy populations.

Thus, while it has been established in large and diverse samples that ES impairments are associated with generalized anxiety, depression, and PTSD symptom severity, less is known about whether personality-linked biases extend to core nonverbal decoding skills like ES. Based on a review of the neural basis of neuroticism and extraversion, there is evidence of individual differences in brain activation in specific regions of the brain during cognitive-affective tasks [19]. Recent work using a cross-sectional fMRI study of 115 healthy adults has connected neuroticism to increased amygdala activity to hippocampal and prefrontal regions during emotional face processing [20], supporting a network-level basis of neuroticism in emotion processing. In prior work examining neuroticism and facial emotion processing using fMRI in 68 healthy participants, activation in the bilateral medial temporal gyrus (MTG) was correlated with higher neuroticism scores [21]. This study also showed that there were no significant sex differences between activation of the MTG and neuroticism scores. In another study of 60 healthy participants, activity and functional amygdala connectivity was measured during an emotional faces gender decision task [22], and results showed that highly neurotic participants display stronger self-referential processing in response to negative emotional faces. A higher degree of neuroticism has been linked to greater sustained medial prefrontal cortex activity when responding to emotional facial expressions [23]. Additionally, increased neuroticism is associated with negative biases in information processing on tasks of emotional categorization and facial expression recognition [24]. While prior work suggests neuroticism biases emotional processing, it is unclear whether these biases extended to core nonverbal decoding skills.

Because neuroticism, a dimension of temperament marked by elevated stress reactivity resulting in the frequent experience of negative affect, is a shared vulnerability factor for each of the disorders previously studied (generalized anxiety, depression, and PTSD) [16,25,26], we would expect that higher levels of neuroticism would be associated with poorer ES performance, but this remains to be tested. As described above, there is evidence that high levels of neuroticism impact emotional facial processing, which can be detected on a neural level [20]. However, because tasks differ between studies, less is known about specific emotion deficits in individuals with varying levels of neuroticism. Thus, the current study tests whether nonverbal decoding is influenced by personality traits using an ES paradigm. While we expected neuroticism to be negatively related to ES performance, we also wanted to explore how extraversion was related to ES, expecting to find a different relationship. Extraversion is linked to positive affect and social engagement [27], which could relate to emotion sensitivity, particularly for positively valenced emotions (happiness). We use extraversion as a trait that is different from neuroticism to explore how ES could be related to personality in general.

College students represent an ideal population for examining the relationship between personality traits and emotion sensitivity. Neuroticism tends to peak in late adolescence and early adulthood, which aligns with the typical age range of undergraduate students [28]. Furthermore, emotional disorders often first emerge during this developmental period [29], making it a key window for studying emotional and personality processes that may influence or be influenced by mental health. Additionally, college students are a population frequently targeted by preventive and clinical interventions, highlighting the relevance of understanding trait-emotion associations in this group [30].

Our study expands on the existing literature in many ways. First, we aim to resolve inconsistencies in emotional facial processing literature by using a relatively novel ES task, the Belmont Emotion Sensitivity Test, which was specifically designed to reduce response bias and allow for comparison of ES across categories (fear, anger, happiness) [15]. Methodological problems in the existing facial emotion processing literature are based on the idea that many tasks conflate discriminability or sensitivity with response bias. Moreover, in traditional facial emotion processing tasks, emotion categories are not comparably difficult. Typically, happiness recognition is easier than detection of other emotions (see [25,26,31]). The Belmont Emotion Sensitivity Task (BEST) corrects for these problems. While prior work has tested ES abilities across the lifespan, in depression, in generalized anxiety symptoms, and in PTSD symptoms, it has not yet linked ES to transdiagnostic risk factors for these disorders, namely neuroticism.

Our primary study hypothesis was that there will be a negative relationship between level of neuroticism and ES abilities across emotions—higher levels of neuroticism will be associated with lower (worse) ES abilities across emotions. We had two additional exploratory hypotheses: first, based on the idea that neuroticism is linked to fear-based disorders (e.g. [ 14,32,33]), we expected fear ES to be most strongly associated with level of neuroticism relative to other emotion categories. Second, we expected that the relationship between extraversion and ES would be positively correlated across emotions based on the idea that a more extraverted temperament would be linked to better social communication [34]. We also examined sex differences in ES as an exploratory analysis.

## Method

This study was reviewed and approved by the Indiana University Institutional Review Board (IRB) as human subjects research (protocol numbers 2011722518 and 12537). All study procedures complied with institutional and federal guidelines for the ethical conduct of research involving human participants. Written informed consent was obtained electronically from all participants prior to study enrollment. Participants were informed that participation was voluntary and that they could withdraw from the study at any time without penalty. The study was not preregistered. Data is available on Open Science Framework, https://osf.io/pvbmc/files/osfstorage. Participants were recruited between March 2021 and September 2023. Participants were sampled from two distinct sources: (1) a psychology student subject pool at a large Midwestern university and (2) a paid study where individuals were recruited through the university online classifieds ads and flyers posted around campus. In the first version, students received course credit for participating in the study. In the second version of the study, participants could earn up to $60 for completing the study. Participants were excluded from analyses if they did not complete the emotion sensitivity tests (*n* = 9), yielding a final analytic sample of 289 participants. Participants completed the study online using an electronic device of their own (any laptop, desktop, smartphone, or tablet).

### Participants

Participants ranged in age from 18 to 56 years (M = 20.46, SD = 4.97). Participants reported both sex assigned at birth and current gender identity. Because gender identity closely aligned with sex (97% concordance), and our analyses focused on group-level differences, we used sex (male/female) as the primary grouping variable. We refer to this variable as *sex* throughout the manuscript. The analytic sample included 69 male and 220 female participants. Available sample sizes for the emotion sensitivity outcomes were slightly smaller because of variable-level missingness (anger: 66 males, 212 females; fear: 65 males, 212 females; happiness: 66 males, 211 females). The majority of participants identified as White (68.49%), followed by Asian (18.49%), Hispanic (5.48%), and Black (4.11%). Most participants were undergraduate students (freshman = 42.47%; sophomore = 20.89%; junior = 15.75%; senior = 10.27%), with a smaller proportion enrolled in graduate school (8.56%).

### Measures

#### *Belmont Emotion Eensitivity Test* (BEST [15];).

The Karolinska Directed Emotional Faces (KDEFS) database [35] was used to create emotional face stimuli. Faces were morphed between any two pairs of angry, happy, and fearful faces, across a set of over two dozen identities. Anger, fear, and happiness sensitivity were each assessed with a separate subtest (anger sensitivity, fear sensitivity, and happiness sensitivity subtests). Each ES subtest shows 56 pairs of faces, one pair at a time, presented on screen at the same time for 1000 milliseconds. Participants were asked to indicate, “Which face is more angry?”, “Which face is more fearful?”, or “Which face is more happy?” to evaluate anger ES, fear ES, and happiness ES, respectively. Trials were ordered with increasing difficulty across three blocks (easy trials = 8, medium trials = 20, and hard trials = 28) for each subtest. The BEST was designed to reduce response bias and more specifically assess sensitivity to differences in emotional intensity. It uses a forced-choice format in which participants indicate which of two simultaneously presented faces shows more of a target emotion. Because the target emotion is fixed within each block and participants are not required to choose among competing emotion labels, the task minimizes category-based response bias that can affect traditional emotion recognition tasks. Thus, BEST more directly indexes discrimination of emotional intensity than tasks requiring categorical labeling of single faces [ 2,36]. Higher scores are indicative of more enhanced emotion sensitivity, or better emotion perception skills. A maximum score of 56 is possible for each of the three emotion categories. Typically, participants would be excluded from analyses if they had less than 50% accuracy (chance performance), however, there were no participants who met this threshold of performance, and thus all participants were included. For more details on BEST development, see [37].

#### *Depression Anxiety and Stress Scales* (DASS [38]).

The Depression Anxiety and Stress Scales (DASS) is a 21-item measure rated on a 4-point Likert scale (0 = “Does not apply to me at all” to 3 = “Applied to me very much, or most of the time”). It assesses levels of depression, general anxiety, and general negative affect/stress symptoms. The DASS has excellent psychometric properties, according to both exploratory and confirmatory factor analyses [ 38,39] . In our sample, internal consistency was excellent (Cronbach’s alpha = .92)

#### *Multidimensional Emotional Disorder Inventory* [40].

The Multidimensional Emotional Disorder Inventory (MEDI) is a relatively novel measure of transdiagnostic dimensions of emotional disorder psychopathology. It consists of 49 items that are rated on a 9-point Likert scale (0 = “Not characteristic of me/does not apply to me” to 8 = “Extremely characteristic of me/applies to me very much”). The initial validation of this measure used factor analysis to determine the factor structure of nine empirically supported transdiagnostic dimensions proposed by Brown & Barlow [41]. These nine dimensions include neurotic temperament, positive temperament, depression, autonomic arousal, somatic anxiety, social anxiety, intrusive cognitions, traumatic reexperiencing, and avoidance. The MEDI showed good evidence of reliability and validity in its initial evaluation [40], and has since been extended to other populations including Spanish clinical samples [42,43]. We used the MEDI neurotic temperament subscale as our measure of neuroticism and negative affectivity and the MEDI positive temperament subscale as our measure of extraversion to capture social reward sensitivity, affective engagement, and enthusiasm. These MEDI dimensions are conceptually aligned with, but not identical to, traditional personality constructs assessed in Big Five measures [44]. This framing is consistent with prior MEDI work in both nonclinical and clinical samples showing adequate reliability and validity for the neurotic temperament and positive temperament dimensions. Higher scores indicate a greater degree of the trait/temperament. In our sample, internal consistency of the MEDI was excellent (Cronbach’s alpha = .93).

### Data analyses

All analyses were conducted in R. Pearson correlations were used to examine associations among personality traits, mood symptoms, and emotion sensitivity scores. We report 95% confidence intervals for correlation coefficients. Sex differences were tested using Welch’s t-tests to account for unequal group sizes and possible heterogeneity of variance; degrees of freedom therefore vary across tests based on the Welch-Satterthwaite approximation and minor variable-level missingness. To reduce Type I error across related sets of tests, Benjamini-Hochberg false discovery rate (FDR) correction was applied within each family of primary analyses. To evaluate the association of personality traits with emotion sensitivity, separate linear regression models were estimated for each emotion sensitivity outcome. Primary regression models included either neuroticism or extraversion as the focal predictor and adjusted for age, sex, depression, anxiety, and stress. Standardized beta coefficients and 95% confidence intervals are reported for the focal predictors.

## Results

ES scores for each emotion category were calculated based on total correct out of 56 face pairs. In our sample, the average happiness score was 45.96 (*SD* = 4.44), average fear score was 44.61 (*SD* = 6.31), and average anger score was 46.70 (*SD* = 5.58). ES scores were correlated with each other, as is typical (Happiness and Anger: *r* = .56, *p* < .001; Happiness and Fear: *r* = .48, *p* < .001; Fear and Anger: *r* = .70, *p* < . 001). The average DASS Depression score in our sample was 9.03 (*SD* = 9.25, range 0–42), indicating a normal to mild level of depression. The average DASS Anxiety score in our sample was 7.99 (*SD* = 7.70, range 0–38), indicating a mild level of anxiety. The average DASS Stress score in our sample was 12.51 (*SD* = 8.55, range 0–42), indicating a normal level of stress. The average MEDI neuroticism score in our sample was 16.53 (*SD* = 8.47, range 0–40), and the average MEDI positive temperament score was 25.20 (*SD* = 6.86, range 2–40).

To assess the distributional properties of each variable, we examined skewness and kurtosis separately for male and female participants. Variables were considered approximately normally distributed if skewness values were between −1 and +1 and kurtosis values were within ±2, following conventional guidelines. Emotion sensitivity scores and personality traits (neuroticism and extraversion) demonstrated acceptable levels of skewness and kurtosis, suggesting normal distributions. In contrast, scores on the DASS depression, anxiety, and stress subscales exhibited moderate positive skew and higher kurtosis in both males and females, indicating non-normal distributions. This pattern is common in non-clinical or student samples, where most individuals endorse low levels of symptoms. Given the large sample size and relative robustness of t-tests, parametric analyses were deemed appropriate.

Correlation analyses showed that neuroticism was negatively associated with extraversion and positively associated with depression, anxiety, and stress. Extraversion was negatively associated with depression and anxiety. In contrast, neither neuroticism nor extraversion was significantly associated with anger, fear, or happiness emotion sensitivity, and all corresponding confidence intervals included zero. Correlation coefficients, 95% confidence intervals, and FDR-adjusted p-values are reported in Table 1.

**Table 1:** Correlations of neuroticism and extraversion with mood symptoms and emotion sensitivity in 289 nonclinical adults.

Predictor	Outcome	n	r	95% CI	p	pFDR
Neuroticism	Extraversion	289	−.14	[-.26, -.03]	.014	.035
	Depression	289	.55	[.46,.62]	<.001	<.001
	Anxiety	289	.63	[.55,.69]	<.001	<.001
	Stress	289	.68	[.61,.74]	<.001	<.001
	ES anger	278	.04	[-.08,.16]	.495	.630
	ES fear	277	.08	[-.04,.20]	.183	.367
	ES happiness	277	−.01	[-.13,.10]	.811	.857
Extraversion	Depression	289	−.45	[-.53, -.35]	<.001	<.001
	Anxiety	289	−.14	[-.25, -.03]	.015	.035
	Stress	289	−.12	[-.23, -.01]	.038	.082
	ES anger	278	.01	[-.10,.13]	.826	.857
	ES fear	277	.01	[-.11,.13]	.888	.888
	ES happiness	277	.07	[-.05,.19]	.246	.406

*Note.* Neuroticism = Multidimensional Emotional Disorder Inventory neuroticism scale; Extraversion = Multidimensional Emotional Disorder Inventory extraversion scale; Depression = Depression Anxiety and Stress Scale (DASS) – Depression Scale; Anxiety = DASS Anxiety Scale; Stress = DASS Stress scale; ES anger = Emotion sensitivity anger scale; ES fear = Emotion sensitivity fear scale; ES happiness = Emotion sensitivity happiness scale. pFDR values reflect Benjamini-Hochberg false discovery rate correction within the correlation family. Confidence intervals are 95% confidence intervals for Pearson correlation coefficients.

We examined sex differences in our sample based on prior research demonstrating that females tend to outperform males on emotion sensitivity tasks [17,18]. Because sex groups were unequal in size and available sample size differed slightly across variables, sex comparisons were conducted using Welch’s t-tests. Females reported significantly higher neuroticism, extraversion, and stress than males. However, males and females did not differ significantly on anger, fear, or happiness emotion sensitivity, see Table 2.

**Table 2:** Group differences in emotion sensitivity, personality, anxiety, and mood scores by sex in a sample of 289 nonclinical adults.

Variable	Male, n	Male M (SD)	Female, n	Female M (SD)	t	df	p	95% CI for mean difference	Cohen’s d
ES anger	66	46.80 (5.70)	212	46.67 (5.60)	0.17	106.85	.868	[-1.45, 1.72]	0.02
ES fear	65	44.00 (6.31)	212	44.77 (6.31)	−0.86	106.17	.392	[-2.54, 1.01]	−0.12
ES happiness	66	45.52 (4.37)	211	46.11 (4.49)	−0.97	111.27	.336	[-1.83, 0.63]	−0.13
Neuroticism	69	12.74 (7.67)	220	17.72 (8.38)	−4.60	122.97	<.001	[-7.12, -2.84]	−0.61
Extraversion	69	23.51 (6.93)	220	25.74 (6.76)	−2.34	111.51	.021	[-4.11, -0.34]	−0.33
Depression	69	8.90 (8.70)	220	9.20 (9.44)	−0.25	122.30	.806	[-2.73, 2.12]	−0.03
Anxiety	69	6.84 (6.93)	220	8.46 (7.90)	−1.64	128.09	.104	[-3.58, 0.34]	−0.21
Stress	69	10.23 (7.40)	220	13.40 (8.70)	−2.97	132.06	.004	[-5.28, -1.06]	−0.38

*Note.* Neuroticism = Multidimensional Emotional Disorder Inventory neuroticism scale; Extraversion = Multidimensional Emotional Disorder Inventory extraversion scale; Depression = Depression Anxiety and Stress Scale (DASS) depression scale; Anxiety = DASS anxiety scale; Stress = DASS stress scale; ES anger = emotion sensitivity anger scale; ES fear = emotion sensitivity fear scale; ES happiness = emotion sensitivity happiness scale. Sex differences were tested using Welch’s t-tests. Degrees of freedom vary across tests because Welch’s t-tests use the Welch-Satterthwaite approximation and because available sample size differed slightly across variables due to missing data. Confidence intervals represent 95% confidence intervals for the mean difference.

**Fig 1:**
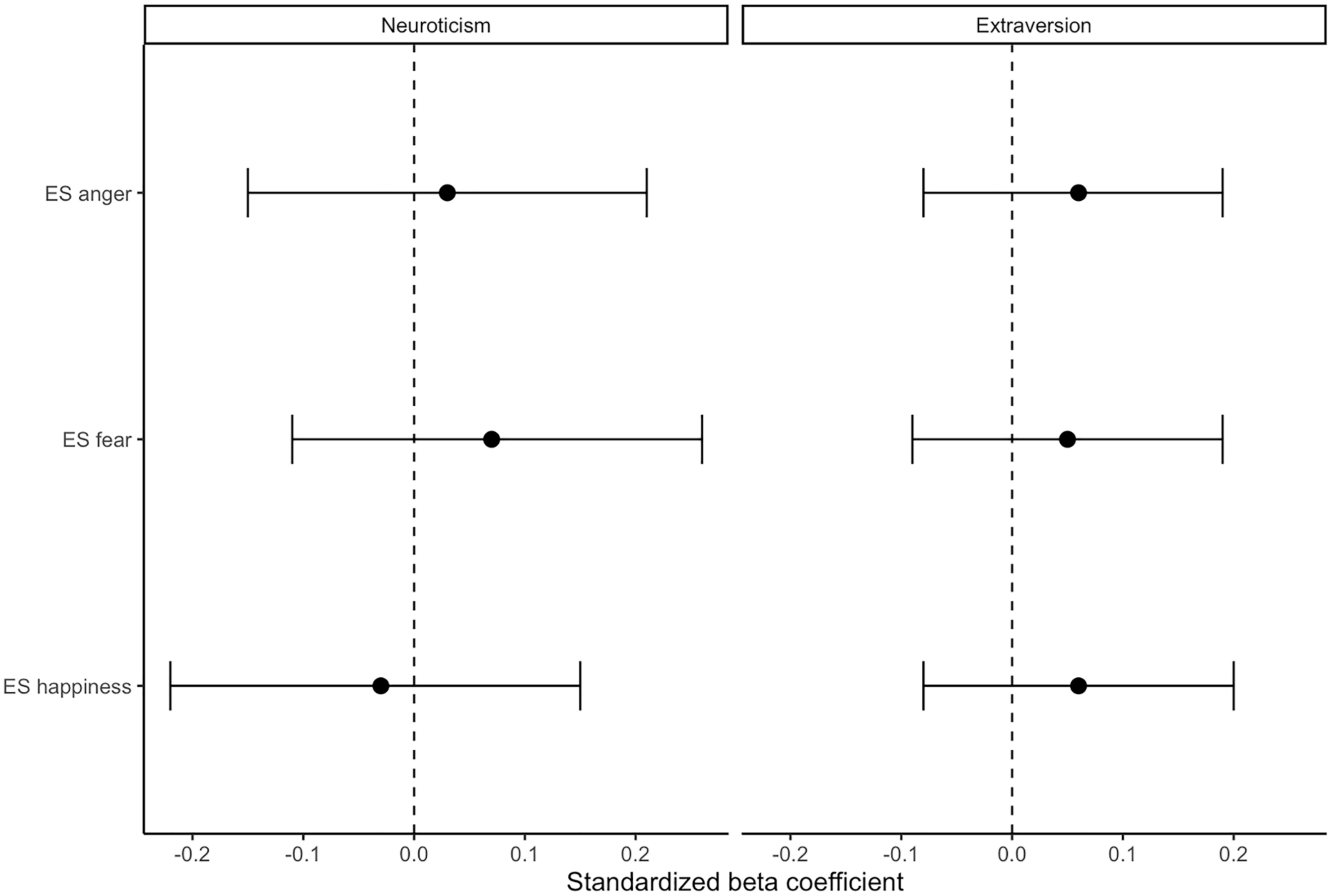
Standardized regression coefficients for associations of neuroticism and extraversion with anger, fear, and happiness emotion sensitivity. Note. Error bars represent 95% confidence intervals. All confidence intervals include zero.

We next conducted multiple linear regressions to evaluate the relationship between neuroticism and emotion sensitivity (ES) scores for anger, fear, and happiness, controlling for sex, age, and anxiety and mood symptoms (depression, anxiety, and stress). In adjusted regression models, neuroticism was not a significant predictor of ES anger (β = .03, 95% CI [−.15,.21], p = .759), ES fear (β = .07, 95% CI [−.11,.26], p = .427), or ES happiness (β = −.03, 95% CI [−.22,.15], p = .708). Similarly, extraversion was not a significant predictor of ES anger (β = .06, 95% CI [−.08,.19], p = .422), ES fear (β = .05, 95% CI [−.09,.19], p = .475), or ES happiness (β = .06, 95% CI [−.08,.20], p = .407). Interpretation was unchanged after FDR correction (all psFDR ≥ .713). None of the full models accounted for substantial variance in performance (adjusted R²s < .01), see Fig 1.

To test whether the relationship between neuroticism and ES varied by sex, we ran moderation models including a neuroticism × sex interaction term. None of the interaction effects were significant (Anger: *β* = −0.09, *p* = .393; Fear: *β* = −0.13, *p* = .255; Happiness: *β* = −0.13, *p* = .123), suggesting that the association between neuroticism and ES did not differ for males versus females. Taken together, these findings suggest that neither neuroticism nor extraversion significantly predict emotion sensitivity in this sample, and that these associations are not moderated by sex.

## Discussion

To the best of our knowledge, this was the first study to examine how levels of neuroticism and extraversion are related to emotion sensitivity. In this sample of predominantly healthy college students, neuroticism and extraversion were not robust predictors of performance on the BEST. These findings suggest that broad personality traits may show limited associations with this specific form of perceptual discrimination of emotional intensity, rather than supporting a broader conclusion that emotion perception is independent of personality. Indeed, results showed that while females had higher levels of neuroticism and extraversion than males in our sample, there were no significant differences in ES performance between males and females, and level of neuroticism and extraversion did not significantly impact ES. These findings suggest weaker associations than some prior work linking personality to nonverbal communication (e.g. [45]). Additionally, the relationship between neuroticism and sex did not moderate ES performance. Personality traits may be less central predictors of ES than demographic or clinical predictors. There are several potential reasons for our pattern of findings, which we discuss below.

Our result that neuroticism was not related to ES abilities could be a function of our sample and task. First, our sample was drawn from generally healthy students at a large university in the Midwest. Additionally, our sample was predominantly female. Traditionally, prior work has shown that females have higher levels of ES abilities for fear [18,46] and higher levels of generalized anxiety symptoms [15]. Our results showed that while females had significantly higher levels of stress, they did not differ significantly in level of anxiety and depression symptoms, which could partially explain why we did not detect differences in ES in our sample. Moreover, while previous studies have reported female advantages in ES (e.g. [12,14,17,47,48]), a possible explanation for why we did not replicate this is reduced variability in our non-clinical sample. Increasing evidence suggests that the magnitude of sex differences in emotional perception may depend on specific emotion categories and contextual demands [25], which may further explain our null findings. Moreover, less is known about whether personality-linked differences in emotional processing extend to perceptual discrimination of emotional intensity [26], as measured by emotion sensitivity, in a nonclinical sample.

Our pattern of findings may be also due to our measurement tools to assess neuroticism and extraversion. The Multidimensional Emotional Disorder Inventory (MEDI) may not be the best tool for capturing varying levels of temperament in college students. It is possible that a different measure of neuroticism, such as the NEO-Five Factor Inventory (NEO-FFI [44]), which is a more widely used measure of personality, may have yielded different results. We opted to use the MEDI because it has excellent psychometric properties and is briefer than the NEO-FFI. Also regarding measurement of neuroticism, it is possible that because we used mean levels of neuroticism while the construct of neuroticism inherently requires emotional variability (fluctuations in negative affect), the metric of quantifying this temperament itself should be reexamined. Recent meta-analytic work suggests that adjusting for the mean is an overcorrection that underestimates the association between neuroticism and negative emotion variability [49]. Future work should examine the connection between neuroticism and emotion dynamics in daily life, as it relates to emotional face processing, emotion regulation, and other related processes.

Furthermore, the relationships between neuroticism, extraversion, and ES may be emotion-specific and require a larger sample to achieve power to detect differences based on level of the trait. For example, Haas and colleagues found a relationship between sad emotional facial expressions and neuroticism when completing an fMRI task, but did not observe this for happy or fearful faces [23]. Prior research suggests that individuals high in neuroticism are particularly attuned to cues of loss, rejection, or distress, which are signals often conveyed by sadness expressions (e.g. [24,31]). In another study, individuals with higher neuroticism had sharper increases in sad mood during a sad mood induction task [37]. In a recent dynamic facial emotional processing study, neuroticism was related to valence of sad emotional face processing [50]. Perhaps examining personality traits and sad emotional expressions would provide more information on the role of trait neuroticism in emotional disorders characterized by sadness, i.e., depression.

There are several limitations of the current work. First, our sample consisted of college students in the Midwest, limiting generalizability. Second, it is possible that there was not enough variation in neuroticism in our sample to observe effects on ES. Our sample was predominantly healthy and thus, there was limited variance in psychopathology and personality traits. In previous research on ES, effect sizes were small, and it is possible that our sample was not powered to detect differences in ES abilities in this nonclinical and range-restricted sample. Null effects may reflect the specific perceptual construct measured by BEST, rather than a general independence of emotion perception from personality traits. It is also possible that device variability [51] or general inattention to the task instructions and test battery may have influenced measurement reliability in the ES task. We did not include an ES task for sadness, as it was not developed at the time of the study. This limits the conclusions about sadness emotion processing as it relates to depression, anxiety, and personality traits. Despite these, there are many strengths and future directions from the current study, including measurement of personality traits and common mental health symptoms in conjunction with measures of ES, as well as our novel tool to assess ES, which was designed to address response bias and discriminability that have impacted consistency across findings in the existing literature on nonverbal emotion decoding. Additionally, while effect sizes were small, they may still hold some informative value for future work including meta-analyses. Future directions include measurement of ES across a broader spectrum of transdiagnostic symptoms, in more severe psychopathology, and with additional measurements of neuroticism and negative emotionality.

In sum, this study examined the relationship between emotion sensitivity for fear, anger, and happiness and neuroticism and extraversion in college students. Contrary to hypotheses, we found no significant relationship between neuroticism and extraversion and ES abilities. We expect that a stronger connection between neuroticism and ES could be observed in a more diverse sample with a higher level of personality characteristics and life stress. We found strong and significant relationships between neuroticism and symptoms of depression and anxiety. We observed significant sex differences in levels of neuroticism, extraversion, and stress, with females showing higher levels of all three. Future research should further validate ES abilities naturalistically in the real world and continue to explore the relationships between neuroticism and nonverbal communication, especially as it may relate to treatment.

## References

[1] Besel LDS, Yuille JC. Individual differences in empathy: The role of facial expression recognition. Personality and Individual Differences. 2010;49(2):107–12. doi: 10.1016/j.paid.2010.03.013

[2] Rutter LA, Norton DJ, Brown TA. The Impact of Self-Reported Depression Severity and Age on Facial Emotion Recognition in Outpatients with Anxiety and Mood Disorders. J Psychopathol Behav Assess. 2019;42(1):86–92. doi: 10.1007/s10862-019-09755-w

[3] Rutter LA, Norton DJ, Brown BS, Brown TA. A Double-Blind Placebo Controlled Study of Intranasal Oxytocin’s Effect on Emotion Recognition and Visual Attention in Outpatients with Emotional Disorders. Cognit Ther Res. 2019;43(3):523–34. doi: 10.1007/s10608-018-9974-x 31130760 PMC6533004

[4] Phillips ML, Drevets WC, Rauch SL, Lane R. Neurobiology of emotion perception I: The neural basis of normal emotion perception. Biol Psychiatry. 2003;54(5):504–14. doi: 10.1016/s0006-3223(03)00168-9 12946879

[5] Davis MH. The effects of dispositional empathy on emotional reactions and helping: A multidimensional approach. Journal of Personality. 1983;51(2):167–84. doi: 10.1111/j.1467-6494.1983.tb00860.x

[6] Mueser KT, Doonan R, Penn DL, Blanchard JJ, Bellack AS, Nishith P, et al. Emotion recognition and social competence in chronic schizophrenia. J Abnorm Psychol. 1996;105(2):271–5. doi: 10.1037//0021-843x.105.2.271 8723008

[7] Attwood AS, Easey KE, Dalili MN, Skinner AL, Woods A, Crick L, et al. State anxiety and emotional face recognition in healthy volunteers. R Soc Open Sci. 2017;4(5):160855. doi: 10.1098/rsos.160855 28572987 PMC5451788

[8] Bradley BP, Mogg K, White J, Groom C, de Bono J. Attentional bias for emotional faces in generalized anxiety disorder. Br J Clin Psychol. 1999;38(3):267–78. doi: 10.1348/014466599162845 10532148

[9] Demenescu LR, Kortekaas R, den Boer JA, Aleman A. Impaired attribution of emotion to facial expressions in anxiety and major depression. PLoS One. 2010;5(12):e15058. doi: 10.1371/journal.pone.0015058 21152015 PMC2995734

[10] Gur RC, Erwin RJ, Gur RE, Zwil AS, Heimberg C, Kraemer HC. Facial emotion discrimination: II. Behavioral findings in depression. Psychiatry Res. 1992;42(3):241–51. doi: 10.1016/0165-1781(92)90116-k 1496056

[11] Fujita BN, Harper RG, Wiens AN. Encoding-decoding of nonverbal emotional messages: Sex differences in spontaneous and enacted expressions. J Nonverbal Behav. 1980;4(3):131–45. doi: 10.1007/bf00986815

[12] Hall JA. Gender effects in decoding nonverbal cues. Psychological Bulletin. 1978;85(4):845–57. doi: 10.1037/0033-2909.85.4.845

[13] Rotter NG, Rotter GS. Sex differences in the encoding and decoding of negative facial emotions. J Nonverbal Behav. 1988;12(2):139–48. doi: 10.1007/bf00986931

[14] Wingenbach TSH, Ashwin C, Brosnan M. Sex differences in facial emotion recognition across varying expression intensity levels from videos. PLoS One. 2018;13(1):e0190634. doi: 10.1371/journal.pone.0190634 29293674 PMC5749848

[15] Rutter LA, Dodell-Feder D, Vahia IV, Forester BP, Ressler KJ, Wilmer JB, et al. Emotion sensitivity across the lifespan: Mapping clinical risk periods to sensitivity to facial emotion intensity. J Exp Psychol Gen. 2019;148(11):1993–2005. doi: 10.1037/xge0000559 30777778

[16] Rutter LA, Scheuer L, Vahia IV, Forester BP, Smoller JW, Germine L. Emotion sensitivity and self-reported symptoms of generalized anxiety disorder across the lifespan: A population-based sample approach. Brain Behav. 2019;9(6):e01282. doi: 10.1002/brb3.1282 30993908 PMC6576169

[17] Rutter LA, Passell E, Scheuer L, Germine L. Depression severity is associated with impaired facial emotion processing in a large international sample. J Affect Disord. 2020;275:175–9. doi: 10.1016/j.jad.2020.07.006 32734904 PMC7428842

[18] Rutter LA, Lind C, Howard J, Lakhan P, Germine L. Posttraumatic stress symptom severity is associated with impaired processing of emotional faces in a large international sample. J Trauma Stress. 2022;35(4):1263–72. doi: 10.1002/jts.22834 35366020 PMC9543058

[19] Canli T. Functional brain mapping of extraversion and neuroticism: learning from individual differences in emotion processing. J Pers. 2004;72(6):1105–32. doi: 10.1111/j.1467-6494.2004.00292.x 15509278

[20] Meiering MS, Weigner D, Grimm S, Enge S. Neuroticism is associated with increased amygdala connectivity to hippocampal and prefrontal regions during emotional face processing. Neuroimage. 2026;325:121655. doi: 10.1016/j.neuroimage.2025.121655 41412407

[21] Klamer S, Schwarz L, Krüger O, Koch K, Erb M, Scheffler K, et al. Association between Neuroticism and Emotional Face Processing. Sci Rep. 2017;7(1):17669. doi: 10.1038/s41598-017-17706-2 29247161 PMC5732281

[22] Cremers HR, Demenescu LR, Aleman A, Renken R, van Tol M-J, van der Wee NJA, et al. Neuroticism modulates amygdala-prefrontal connectivity in response to negative emotional facial expressions. Neuroimage. 2010;49(1):963–70. doi: 10.1016/j.neuroimage.2009.08.023 19683585

[23] Haas BW, Constable RT, Canli T. Stop the sadness: Neuroticism is associated with sustained medial prefrontal cortex response to emotional facial expressions. Neuroimage. 2008;42(1):385–92. doi: 10.1016/j.neuroimage.2008.04.027 18511299 PMC2789588

[24] Chan SWY, Goodwin GM, Harmer CJ. Highly neurotic never-depressed students have negative biases in information processing. Psychol Med. 2007;37(9):1281–91. doi: 10.1017/S0033291707000669 17493298

[25] Porter S, ten Brinke L, Wallace B. Secrets and Lies: Involuntary Leakage in Deceptive Facial Expressions as a Function of Emotional Intensity. J Nonverbal Behav. 2011;36(1):23–37. doi: 10.1007/s10919-011-0120-7

[26] Ringwald WR, Wright AGC. Overcoming the confound of means and variability for measuring everyday emotion dynamics related to neuroticism. https://osf.io/m68qv/

[27] Watson D, Clark LA, McIntyre CW, Hamaker S. Affect, personality, and social activity. J Pers Soc Psychol. 1992;63(6):1011–25. doi: 10.1037//0022-3514.63.6.1011 1460554

[28] Hisler GC, Krizan Z, DeHart T, Wright AGC. Neuroticism as the intensity, reactivity, and variability in day-to-day affect. Journal of Research in Personality. 2020;87:103964. doi: 10.1016/j.jrp.2020.103964

[29] Xiao H, Carney DM, Youn SJ, Janis RA, Castonguay LG, Hayes JA, et al. Are we in crisis? National mental health and treatment trends in college counseling centers. Psychol Serv. 2017;14(4):407–15. doi: 10.1037/ser0000130 29120199

[30] Nyer M, Farabaugh A, Fehling K, Soskin D, Holt D, Papakostas GI, et al. Relationship between sleep disturbance and depression, anxiety, and functioning in college students. Depress Anxiety. 2013;30(9):873–80. doi: 10.1002/da.22064 23681944 PMC3791314

[31] Olvet DM, Hajcak G. The error-related negativity relates to sadness following mood induction among individuals with high neuroticism. Soc Cogn Affect Neurosci. 2012;7(3):289–95. doi: 10.1093/scan/nsr007 21382967 PMC3304478

[32] Clark LA, Watson D. Distress and fear disorders: an alternative empirically based taxonomy of the “mood” and “anxiety” disorders. Br J Psychiatry. 2006;189:481–3. doi: 10.1192/bjp.bp.106.03825 17139030

[33] Griffith JW, Zinbarg RE, Craske MG, Mineka S, Rose RD, Waters AM, et al. Neuroticism as a common dimension in the internalizing disorders. Psychol Med. 2010;40(7):1125–36. doi: 10.1017/S0033291709991449 19903363 PMC2882529

[34] Li J, Tian M, Fang H, Xu M, Li H, Liu J. Extraversion predicts individual differences in face recognition. Commun Integr Biol. 2010;3(4):295–8. doi: 10.4161/cib.3.4.12093 20798810 PMC2928302

[35] Lundqvist D, Flykt A, Ohman A. The Karolinska directed emotional faces (KDEF). CD ROM from Department of Clinical Neuroscience, Psychology section, Karolinska Institutet. 1998.

[36] Macmillan NA, Creelman CD. Detection Theory: A User's Guide (2nd ed.). Psychology Press;2004. 10.4324/9781410611147

[37] Moshirian Farahi SM, Asghari Ebrahimabad MJ, Gorji A, Bigdeli I, Moshirian Farahi SMM. Neuroticism and Frontal EEG Asymmetry Correlated With Dynamic Facial Emotional Processing in Adolescents. Front Psychol. 2019;10:175. doi: 10.3389/fpsyg.2019.00175 30800085 PMC6375848

[38] Lovibond PF, Lovibond SH. The structure of negative emotional states: comparison of the Depression Anxiety Stress Scales (DASS) with the Beck Depression and Anxiety Inventories. Behav Res Ther. 1995;33(3):335–43. doi: 10.1016/0005-7967(94)00075-u 7726811

[39] Brown TA, Chorpita BF, Barlow DH. Structural relationships among dimensions of the DSM-IV anxiety and mood disorders and dimensions of negative affect, positive affect, and autonomic arousal. J Abnorm Psychol. 1998;107(2):179–92. 10.1037//0021-843x.107.2.179 9604548

[40] Rosellini AJ, Brown TA. The Multidimensional Emotional Disorder Inventory (MEDI): Assessing transdiagnostic dimensions to validate a profile approach to emotional disorder classification. Psychol Assess. 2019;31(1):59–72. doi: 10.1037/pas0000649 30160498 PMC6312761

[41] Brown TA, Barlow DH. A proposal for a dimensional classification system based on the shared features of the DSM-IV anxiety and mood disorders: implications for assessment and treatment. Psychol Assess. 2009;21(3):256–71. doi: 10.1037/a0016608 19719339 PMC2845450

[42] Osma J, Martínez-Loredo V, Quilez-Orden A, Peris-Baquero O, Ferreres-Galán V, Prado-Abril J, et al. Multidimensional emotional disorders inventory: Reliability and validity in a Spanish clinical sample. J Affect Disord. 2023;320:65–73. doi: 10.1016/j.jad.2022.09.140 36183816

[43] Osma J, Martínez-Loredo V, Quilez-Orden A, Peris-Baquero Ó, Suso-Ribera C. Validity Evidence of the Multidimensional Emotional Disorders Inventory among Non-Clinical Spanish University Students. Int J Environ Res Public Health. 2021;18(16):8251. doi: 10.3390/ijerph18168251 34444001 PMC8392424

[44] Costa PT, McCrae RR. The Revised NEO Personality Inventory (NEO-PI-R). The SAGE Handbook of Personality Theory and Assessment: Volume 2 — Personality Measurement and Testing. SAGE Publications Ltd. 2008. p. 179–98. doi: 10.4135/9781849200479.n9

[45] Kret ME, Ploeger A. Emotion processing deficits: a liability spectrum providing insight into comorbidity of mental disorders. Neurosci Biobehav Rev. 2015;52:153–71. doi: 10.1016/j.neubiorev.2015.02.011 25725415

[46] McClure EB. A meta-analytic review of sex differences in facial expression processing and their development in infants, children, and adolescents. Psychol Bull. 2000;126(3):424–53. doi: 10.1037/0033-2909.126.3.424 10825784

[47] Hall JA, Matsumoto D. Gender differences in judgments of multiple emotions from facial expressions. Emotion. 2004;4(2):201–6. doi: 10.1037/1528-3542.4.2.201 15222856

[48] Thompson AE, Voyer D. Sex differences in the ability to recognise non-verbal displays of emotion: a meta-analysis. Cogn Emot. 2014;28(7):1164–95. doi: 10.1080/02699931.2013.875889 24400860

[49] Kujawa A, Hajcak G, Torpey D, Kim J, Klein DN. Electrocortical reactivity to emotional faces in young children and associations with maternal and paternal depression. J Child Psychol Psychiatry. 2012;53(2):207–15. doi: 10.1111/j.1469-7610.2011.02461.x 21895650 PMC3522574

[50] Passell E, Strong RW, Rutter LA, Kim H, Scheuer L, Martini P, et al. Cognitive test scores vary with choice of personal digital device. Behav Res Methods. 2021;53(6):2544–57. doi: 10.3758/s13428-021-01597-3 33954913 PMC8568735

[51] Lund HG, Reider BD, Whiting AB, Prichard JR. Sleep patterns and predictors of disturbed sleep in a large population of college students. J Adolesc Health. 2010;46(2):124–32. doi: 10.1016/j.jadohealth.2009.06.016 20113918

